# Acute Effects of Modafinil on Brain Resting State Networks in Young Healthy Subjects

**DOI:** 10.1371/journal.pone.0069224

**Published:** 2013-07-25

**Authors:** Roberto Esposito, Franco Cilli, Valentina Pieramico, Antonio Ferretti, Antonella Macchia, Marco Tommasi, Aristide Saggino, Domenico Ciavardelli, Antonietta Manna, Riccardo Navarra, Filippo Cieri, Liborio Stuppia, Armando Tartaro, Stefano L. Sensi

**Affiliations:** 1 Department of Neuroscience and Imaging, University “G. d’Annunzio” Chieti-Pescara, Italy; 2 Molecular Neurology Unit, Center of Excellence on Aging, University “G. d’Annunzio” Chieti-Pescara, Italy; 3 Department of Psychological sciences, University “G. d’Annunzio” Chieti-Pescara, Italy; 4 School of Engineering, Architecture, and Motor Science, Kore University of Enna, Enna, Italy; 5 Departments of Neurology and Pharmacology, University of California-Irvine, Irvine, California, United States of America; 6 Institute for Mind Impairments and Neurological Disorders, University of California-Irvine, Irvine, California, United States of America; The University of Melbourne, Australia

## Abstract

**Background:**

There is growing debate on the use of drugs that promote cognitive enhancement. Amphetamine-like drugs have been employed as cognitive enhancers, but they show important side effects and induce addiction. In this study, we investigated the use of modafinil which appears to have less side effects compared to other amphetamine-like drugs. We analyzed effects on cognitive performances and brain resting state network activity of 26 healthy young subjects.

**Methodology:**

A single dose (100 mg) of modafinil was administered in a double-blind and placebo-controlled study. Both groups were tested for neuropsychological performances with the Raven’s Advanced Progressive Matrices II set (APM) before and three hours after administration of drug or placebo. Resting state functional magnetic resonance (rs-FMRI) was also used, before and after three hours, to investigate changes in the activity of resting state brain networks. Diffusion Tensor Imaging (DTI) was employed to evaluate differences in structural connectivity between the two groups. Protocol ID: Modrest_2011; NCT01684306; http://clinicaltrials.gov/ct2/show/NCT01684306.

**Principal Findings:**

Results indicate that a single dose of modafinil improves cognitive performance as assessed by APM. Rs-fMRI showed that the drug produces a statistically significant increased activation of Frontal Parietal Control (FPC; p<0.04) and Dorsal Attention (DAN; p<0.04) networks. No modifications in structural connectivity were observed.

**Conclusions and Significance:**

Overall, our findings support the notion that modafinil has cognitive enhancing properties and provide functional connectivity data to support these effects.

**Trial Registration:**

ClinicalTrials.gov NCT01684306 http://clinicaltrials.gov/ct2/show/NCT01684306.

## Introduction

Modafinil (Provigil), a drug on the market since 1997, is employed for the treatment of narcolepsy and other sleep disorders [1], [2]. In recent years, modafinil has also been used off-label to treat cognitive dysfunction in psychiatric disorders such as schizophrenia and the Attention Deficit/Hyperactivity Disorder (ADHD) [3]–[5].

Modafinil is involved in the modulation of orexin, a hypothalamic neuropeptide [6] that regulates wakefulness. Several studies have also indicated that the drug interferes with the activity of additional neurotransmitters like hypocretin, histamine [7], gamma-aminobutyric acid (GABA) [8], glutamate [9], and norepinephrine [1]. Finally, recent studies have shown that modafinil can also block the dopamine transporter (DAT1), thereby increasing brain dopamine levels [10].

The employment of psychostimulants to promote cognitive enhancement has been recently widely debated [11]. Among the drugs that have gathered some interest as cognitive enhancers, modafinil has emerged as a potential pharmacological aid to enhance performance in domains like attention and memory [12]–[14]. However, to date, the potential of this drug as modulator of fluid intelligence (Gf) is still unknown. In this study, we aimed at filling this knowledge gap and evaluated effects of a single dose of modafinil on Gf performances in a cohort of healthy young individuals.

Several amphetamine-like drugs have been employed as cognitive enhancers but all of them have important side effects and show great risks of inducing addiction [15]. Modafinil might have therapeutic potentials compared to other stimulants like methylphenidate [16] and amphetamine [17] as the drug has been reported to produce fewer side effects and shows less risk of inducing addiction. However, this notion has been eventually challenged given the strong effect of modafinil on the dopaminergic system [10].

In this study a single dose (100 mg) of modafinil was administered to healthy young individuals and acute effects on cognition, modulation of brain resting state network (RSNs) activity, and structural connectivity were evaluated. We tested modafinil effects on Gf in a population of young healthy subjects. The study meant to further our knowledge on the activity of the drug in a physiological setting. We chose a relatively low dosage in order to evaluate potential positive effects while reducing at minimum the drug side-effects. This is in line with previous investigations employing the same dosage [14].

Cognition was evaluated in terms of Gf. Gf is a complex human ability that allows flexible thinking, comprehension of abstract relations, and plastic adaptation to new cognitive problems, situations or events [18]. Gf is considered a major factor in affecting learning and usually investigated with the Raven’s advanced progressive matrices II set (APM) [19]. Upon APM evaluation, subjects are asked to choose missing parts of visuo-spatial patterns (i.e., matrices) in a set of fixed alternatives. The tasks involve flexibility in thinking, pattern matching abilities as well as relational reasoning [18].

Resting state fMRI (rs-fMRI) is an excellent tool to evaluate modifications of functional connectivity [20]. The technique has emerged as an important modality of fMRI acquisition. Compared to task-related fMRI, rs-FMRI offers some advantages. Rs-fMRI allows the simultaneous investigation of multiple cortical circuits at once. The possibility of studying subjects at rest greatly reduces confounding factors like inter-individual variability in task compliance and/or performance during fMRI acquisition [21]. For these reasons we decided to employ rs-FMRI to study effects on functional connectivity.

Early rs-fMRI studies have investigated the activity of specific cerebral regions at rest [22] with blood oxygen level dependent (BOLD) fMRI. Rest activity is organized in multiple and highly specific functional RSNs [23]. To date at least ten RSNs have been identified [23]–[26]. Of these ten, the most studied are: the Default Mode Network (DMN) [27]; the Salience Network (SN); the Fronto Parietal Control (FPC) network (lateralized in both hemispheres); the primary Sensory Motor Network (SMN), the Exstrastriate Visual System (EsV), and the Dorsal Attention Network (DAN) [23]. These are the RSNs we chose to investigate as previous studies indicated their relation to the main cognitive domains that encompass Gf [28]–[33].

Diffusion Tensor Imaging (DTI) allows the study of white matter tract integrity and provides complementary information to evaluate structural connectivity [34]. In this study, we have employed DTI to verify if connectivity differences were present at baseline between the two study groups.

## Materials and Methods

### Population study and design

**Figure 1:**
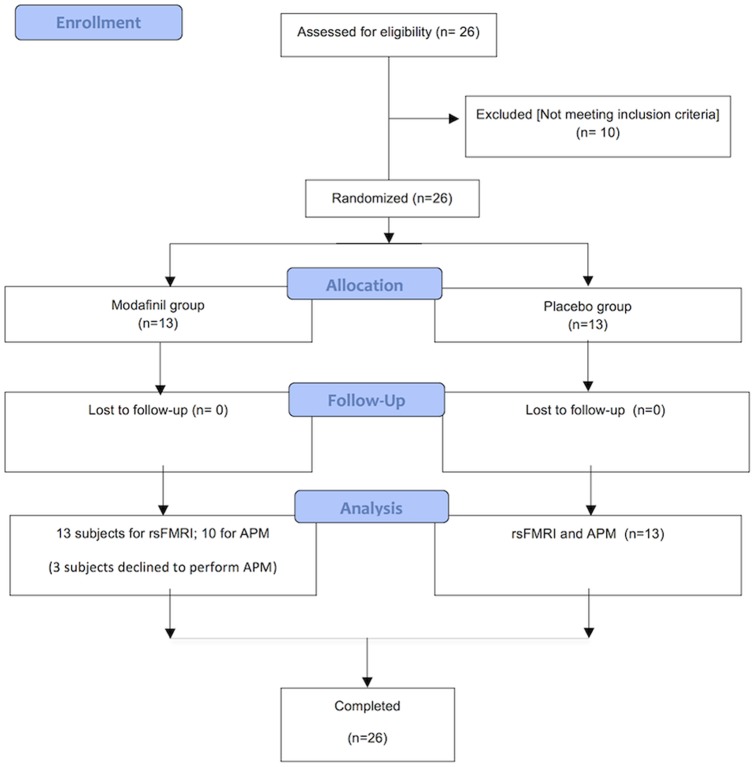
CONSORT Diagram. Flow diagram graphically describes the design of the study: enrollment, intervention, follow-up and data analysis.

The study was approved by our Research and Ethics Committee and written informed consent was obtained from all subjects. All procedures were conducted in accordance to the principles expressed in the Declaration of Helsinki. We enrolled twenty six young male right-handed (as assessed by the Edinburgh Handedness inventory) [35] adults (age range: 25–35 y.o.) with comparable levels of education (13 years). All subjects had no past or current signs of psychiatric, neurological or medical (hypertension, cardiac disorders, epilepsy) conditions as determined by the Millon test and by clinical examination. Subjects showing visual or motor impairments were excluded as well as individuals taking psychoactive drugs or having a history of alcohol abuse. All volunteers were instructed to maintain their usual amount of nicotine and caffeine intake and avoid alcohol consumption in the 12 h before the initiation of the study. Study subjects received, in a double blind fashion, either a single dose of modafinil (modafinil group) or a placebo (placebo group) pill identical to the drug (Figure 1). The day after drug/placebo assumption, subjects were asked about perceived side effects and, in particular, sleep disturbances. All but one reported no modafinil-induced side effects or alterations in the sleep-awake cycle.

### Neuropsychological evaluation and statistical analysis

Subjects performed APM [19] to evaluate Gf [36], before and three hours after modafinil or placebo administration.

APM are commonly employed to evaluate abstract reasoning and considered a useful tool to measure Gf. Gf is defined as basic reasoning skill that is not affected by cultural and education factors. APM items are matrices of figures arranged in three rows and three columns and placed in sequence. Participants are asked to identify missing segments and choose among eight alternative answers. The test consists of four series of matrices. The first series is introductory, made of 12 items, not computed, and used as trial for the following items. The other series consist of 12 items each (for a total of 36). In these series items are placed in order of increased difficulty and presented as A, B, and C [19] [37]. Therefore, we analyzed APM results considering three scores that were based on difficulty levels (A = low, B = medium, C = high) in order to have more detailed information on performances.

Participants completed APM before (pre-test) and after (post-test) rs-fMRI scans and drug/placebo intake. Three subjects in the modafinil group declined to be studied with APM. Measurement of cognitive ability was extrapolated by taking in consideration the number of correct answers. Items were divided in three categories accordingly to the degree of difficulty. Each category was composed of 12 items. Three factor mixed design ANOVA followed by Duncan's post-hoc test was performed and the general linear model (GLM) approach employed after aligned rank transformation (ART) of data [38]. Group (modafinil or placebo) was the between-subjects factor. Time (pre- and post-test), and difficulty levels (low, medium, and high) were the within-subjects factors. Type 1 error (α) for null-hypothesis rejection was set at p<0.050. Statistical analysis was performed using Statistica 6.0 (Statsoft, Tulsa, OK) software.

### Rs-fMRI acquisition

Rs-fMRI BOLD data were separated in three runs lasting four minutes each followed by high resolution T1 anatomical images. Subjects were asked to relax while fixating the central point in the middle of a grey-background screen that was projected on a LCD screen and viewed through a mirror placed above the subject head. Subject head was positioned within an eight-channel coil and foam padding was employed to minimize involuntary head movements. BOLD functional imaging was performed with a Philips Achieva 3T Scanner (Philips Medical Systems, Best, The Netherlands), using T2*-weighted echo planar imaging (EPI) free induction decay (FID) sequences and applying the following parameters: TE 35 ms, matrix size 64×64, FOV 256 mm, in-plane voxel size 4×4 mm, flip angle 75°, slice thickness 4 mm and no gaps. 140 functional volumes consisting of 30 transaxial slices were acquired per run with a volume TR of 1671 ms. High resolution structural images were acquired at the end of the three rs-fMRI runs through a 3D MPRAGE sequence employing the following parameters: sagittal, matrix 256×256, FOV 256 mm, slice thickness 1 mm, no gaps, in-plane voxel size 1 mm×1 mm, flip angle 12°, TR = 9.7 ms and TE = 4 ms. Image data processing was carried out using the BrainVoyager QX software (Brain Innovation, Maastricht, The Netherlands).

RSNs were investigated by means of independent component analysis (ICA) (the protocol for this trial and supporting CONSORT checklist are available as supporting information; see Checklist S1 and Protocol S1). Briefly, independent components (IC) were extracted for each data set and scaled to spatial z-score maps. In each IC map, the z-score value associated to a given voxel reflects the weight of IC time course with respect to its relative measured BOLD data, thereby providing an indirect indication of functional connectivity. Group IC maps, representing the most physiologically relevant and consistently reported RSNs, were obtained (S1). The group map of each network was threshold at a significance level of p = 0.05 (Bonferroni corrected for multiple comparisons). This threshold map was then employed to create a mask of voxels representing the whole network. Distinct masks representing the different nodes of the RSN were also obtained considering the 200 most significant voxels around each local Z-score maximum. For each mask, we then extracted the 52 Z-score values representing individual levels of connectivity in the whole network and in single nodes during different experimental conditions. Individual Z-scores in each node were compared by means of a mixed design ANOVA with the factors group (drug, placebo) and time (pre, post) in order to evaluate statistically significant connectivity changes due to treatment (modafinil or placebo). These ANOVAs were followed by Duncan’s post-hoc tests with Bonferroni correction for multiple comparisons. A Bonferroni correction with a n =  number of nodes  = 22 (see results) was considered to avoid false positives. Statistical significance was set at p<0.050. Statistical analysis was performed using Statistica 6.0 software.

Group-level t-maps resulting from direct voxel-by-voxel contrasts between pre and post-drug conditions were also produced. These maps were threshold at p = 0.05, corrected for multiple comparisons using a cluster size algorithm (S1).

### DTI imaging and analysis

DTI images were acquired before and after drug consumption (exactly 3 h later) and data used to build Fractional Anisotropy (FA) maps. DTI images were acquired using the manufacturer’s diffusion weighted multi slice spin echo EPI pulse sequence with enhanced gradient mode and 16 gradient directions. Image parameters were as follow: field of view, 22.4 cm; slice thickness, 2 mm; imaging matrix, 112×112; repetition time, 10702 ms; echo time, 55 ms; bandwidth in EPI frequency direction, 2.97 kHz; number of slices, 60; slice gap, 0 mm; b-value 800 s/mm2. EPI factor, 59; SENSE factor, 2. For Tract-Based Spatial Statistics (TBSS) analysis, diffusion data were processed using FMRIB’s FSL 4.1.8 toolbox (http://www.fmrib.ox.ac.uk/fsl) [39]. Diffusion-weighted images from scanner were converted to NifTi (http://nifti.nimh.nih.gov/) compressed format using dcm2nii tool from MRIcron (http://www.cabiatl.com/mricro/mricron/). A in-house adapted MATLAB (Mathworks Inc. Natwick, MA) code of DTI Gradient Table Creator [40] was used to align gradient schemes to subject position. Eddy current and motion corrections were carried out using b0 as reference [41] then gradient table corrections were applied [42]. Brain extraction and masking were performed using FSL’s BET. Finally, DTIFIT in FSL’s FDT toolbox [43] was used to fit diffusion tensors to each voxel and compute FA maps for each subject [44]. Standard TBSS pipeline, with n = 50000 (i.e. p = 0.0500+/−0.0019), was applied to gather results.

## Results

### Neuropsychological performances

A mixed design ANOVA revealed that pre-test scores at the three difficulty levels of the task were homogeneous in the two study groups (p = 0.697, 0.651, and 0.552, respectively). In the drug group, mean pre-test scores were 10 (S.D. = 3), 8 (S.D. = 3) and 3 (S.D. = 3) for the low, medium and high level of APM difficulty, respectively. In the placebo group, mean pre-test scores were 11.15 (S.D. = 1.72), 8.69 (S.D. = 2.59), and 4.30 (S.D. = 2.89) for the low, medium and high level of APM difficulty, respectively. We also found that the level of APM difficulty was significant (p<0.001), thereby indicating that performances were worst with items of greater difficulty in both study groups. In contrast, we did not find significant effects of the group (modafinil and placebo) on pre- and post-test scores (p = 0.412). We also did not find significant interactions between the investigated factors.

**Figure 2:**
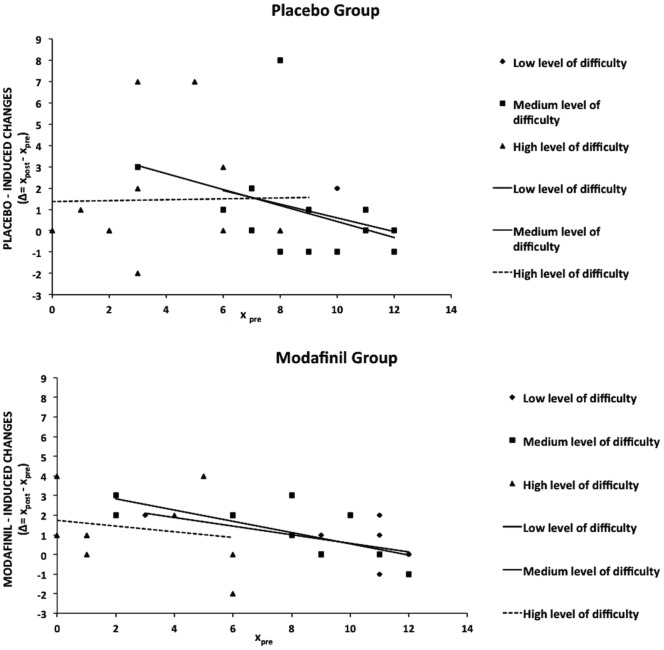
Effects of modafinil on Raven’s advanced progressive matrices II set (APM) performances. Dispersion diagrams for placebo and modafinil. Graphs depict degrees of improvement (expressed as Δ = x_post_-x_pre_; x_post_ =  post test score, x_pre_ =  pre-test score) against pre-test scores (x_pre_) for each level of APM difficulty (low, medium, and high). Segments indicate calculated linear regressions and geometric symbols depict levels of difficulty (low: diamonds; medium: squares; high: triangles).

In order to evaluate individual degrees of modification in APM performances, we calculated differences between post-test and pre-test scores (Δ_τ_ = x_post,i_-x_pre,I_; x_post,i_ = post-test score and x_pre,i_ =  post-test score for the i-th subject) for each study subject when evaluated in the three APM categories (low, medium and high). The higher the difference between post-test and pre-test scores, the greater the improvement. We then performed linear regression analyses in which, for both groups, the dependent variable of the linear model was Δ while the independent variable was the pre-test score. The standardized coefficient of the pendency (β) of the linear model showed a trend toward significance [β = −0.612, t(1,8) = −2.187 p = 0.060] for the drug group in APM scores of the low difficulty level (Figure 2, diamonds). β was statistically significant for the placebo group (β = −0.598, t(1,11) = −2.472 p = 0.031] when studied for the same APM category (low difficulty; Figure 2, diamonds). When considering the APM subset of medium difficulty level, we found that β (−0.703) was statistically significant [t(1,8) = −2.800, p = 0.023] in the drug group (Figure 2, squares) while β (−0.395) of the placebo group was not significant [t(1,11) = −1.426, p = 0.182] (Figure 2, squares). Finally, for APM scores of high difficulty, β was −0.208 for the drug group and −0.023 for the placebo group, respectively. Both values failed to reach statistical significance [t(1,8) = −0.602 p = 0.564], and (t(1,11) = −0.075 p = 0.941, respectively) (Figure 2, triangles).

### Brain functional connectivity

**Figure 3:**
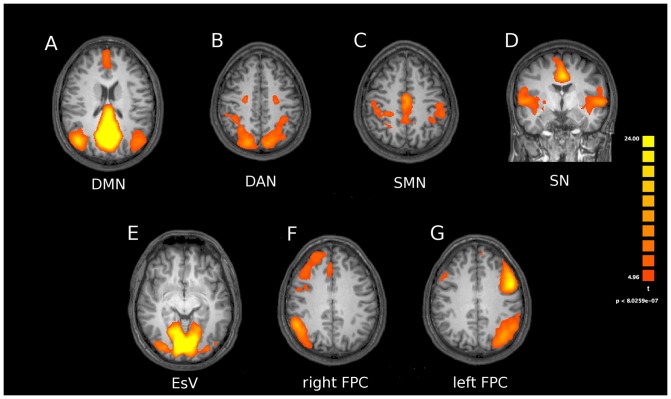
Resting state networks obtained from ICA. Resting state networks obtained with ICA when pooling together groups (modafinil and placebo) and conditions (pre and post treatments). Statistical maps are threshold at p<0.05 (Bonferroni corrected) and overlaid on the Talairach template. Pictures are depicted in radiological convention. DMN: default mode network; DAN: dorsal attention network; SMN: sensorymotor network; SN: salience network; EsV: extrastriate visual; FPC: frontoparietal control network.

We analyzed seven canonical RSNs [25]. These included: (i) the DMN (a network that encompasses the posterior cingulate cortex, the bilateral inferior parietal lobules, and the medial frontal cortex); (ii) the EsV (a network that encompasses the two retinotopic occipital cortices); (iii) the FPC (a network composed by two different subnetworks, lateralized in left and right hemispheres, and encompassing the two intra-parietal cortices and the superior-lateral frontal cortex); (iv) the SMN (a network that includes bilaterally the pre- and post-central gyri, the medial frontal gyrus, corresponding to the primary somatomotor areas and the supplementary motor area), (v) the DAN (a network that includes the bilateral intraparietal sulcus and frontal eye fields), (vi) the SN ( a network including the temporo-insular and anterior cingulate cortex). The RSN group maps are depicted in Figure 3.

**Figure 4:**
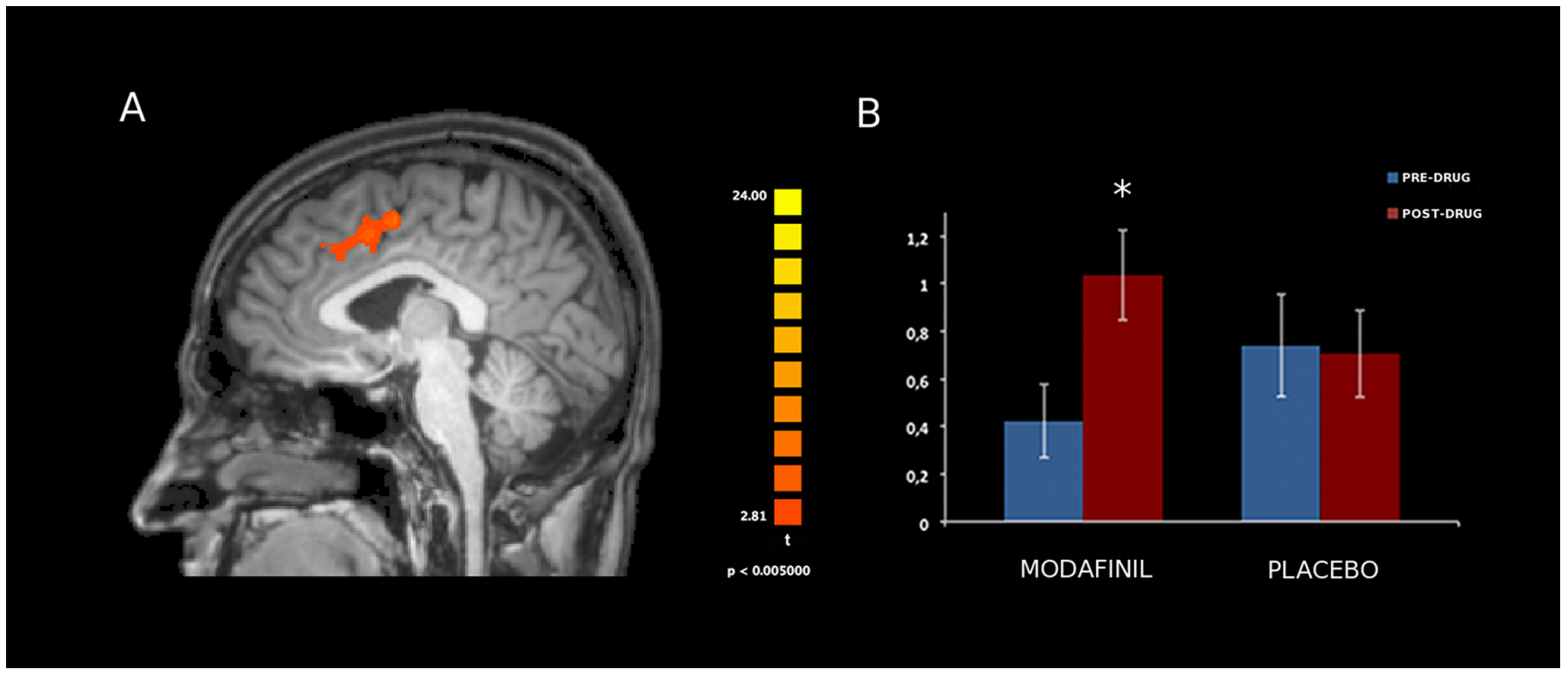
Effects of modafinil on functional connectivity of the left Fronto Parietal Control (lFPC) network. Panel A depicts modafinil-induced changes in connectivity (voxel-by-voxel contrast between pre and post-drug conditions; p<0.05, corrected for multiple comparisons using a cluster size algorithm) in the lFPC network. The picture is shown in radiological convention. Panel B: asterisk indicates significant differences as obtained with the Duncan’s test. Error bars show standard errors. Note the statistically significant increased ACC activity in the modafinil group.

**Figure 5:**
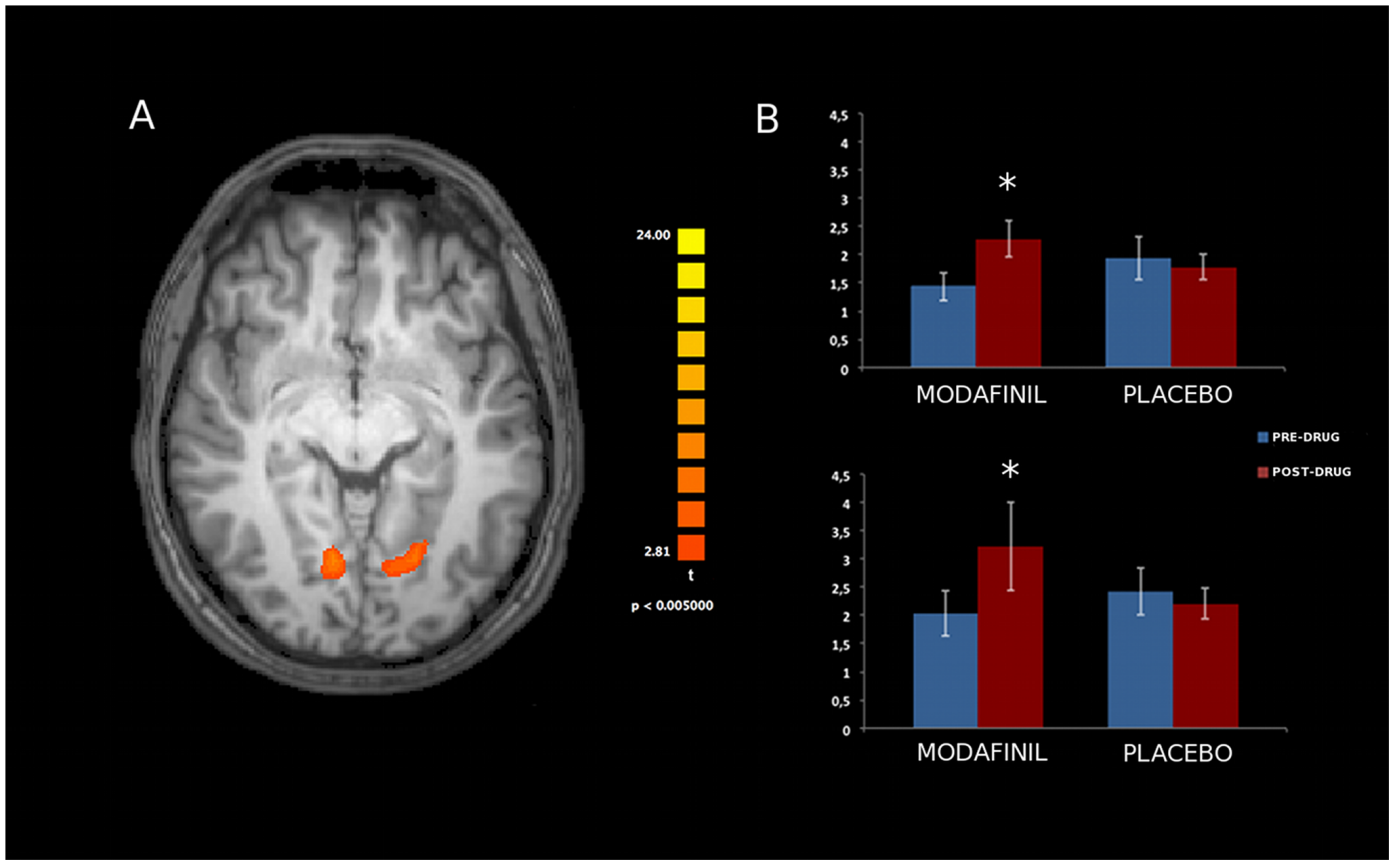
Effects of modafinil on functional connectivity of the Extra striate Visual (EsV) network. Panel A depicts modafinil-induced changes in connectivity (voxel-by-voxel contrast between pre and post-drug conditions, p<0.05, corrected for multiple comparisons using a cluster size algorithm) in EsV network. The picture is shown in radiological convention. Panel B: asterisks indicate significant differences as obtained with the Duncan’s test. Error bars show standard errors. Note the statistically significant increased bilateral occipital pole activity in the modafinil group.

**Figure 6:**
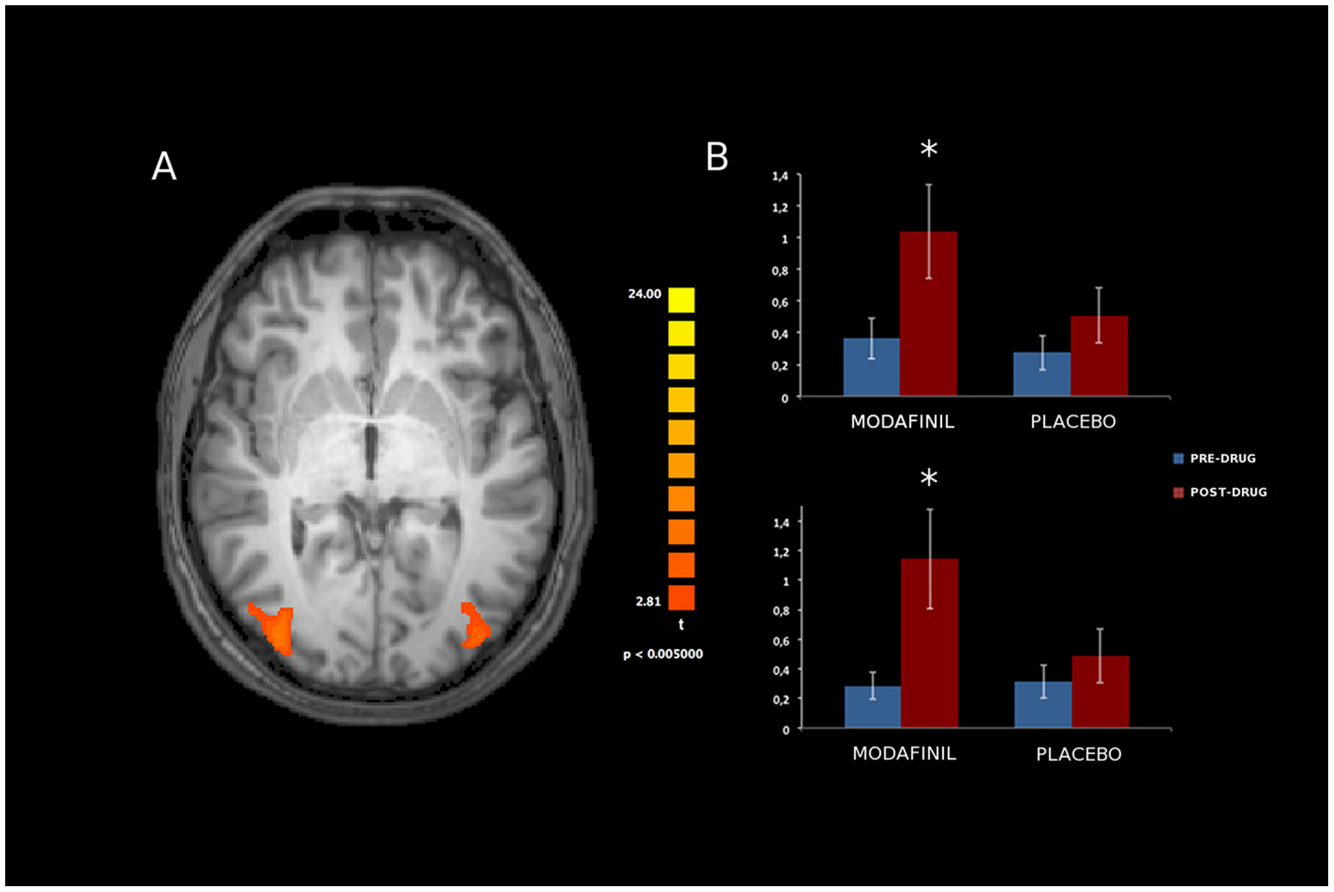
Effects of modafinil on functional connectivity of the Dorsal Attention Network (DAN). Panel A depicts modafinil-induced changes in connectivity (voxel-by-voxel contrast between pre and post-drug conditions, p<0.05, corrected for multiple comparisons using a cluster size algorithm) in the DAN. The picture is shown in radiological convention. Panel B: asterisks indicate significant differences as obtained with the Duncan’s test. Error bars show standard errors. Note the statistically significant increased bilateral occipito-parietal junction activity in the modafinil group.

Analysis of resting state activity of the seven investigated networks [DMN, SN, FPC (right and left components), SMN, EsV, DAN; Figure 3] showed significant modafinil-induced connectivity changes in specific areas of the FPC, EsV, and DAN. Mixed design ANOVA showed a significant group x time interaction in these nodes, thereby revealing, when comparing pre- and post-drug (modafinil) sessions, increased connectivity in the Anterior Cingulate Cortex (ACC) node of the left Frontal Parietal Control (lFPC) network (p<0.002, Duncan’s post-hoc test; Figure 4), in the occipital pole nodes of bilateral EsV (p<0.003, Duncan’s post-hoc test; Figure 5) and in the occipito-parietal junction nodes of bilateral DAN (p<0.002, Duncan’s post-hoc test; Figure 6). No significant differences in functional connectivity were observed when comparing baseline values of the two groups and in placebo group pre/post treatment. Further analysis with multiple comparisons with Bonferroni correction showed statistically significant connectivity changes in the ACC (lFPC; corrected alpha value of p<0.044, Figure 4) and the bilateral occipito-parietal junction (DAN; corrected alpha value of p<0.044, Figure 6) while a trend toward significance (p<0.067, corrected) was observed in bilateral occipital pole nodes (EsV; Figure 5). Pearson correlation analysis between ICA z-scores and behavioural data did not show significant effects in the investigated nodes.

Group-level t-maps resulting from direct voxel-by-voxel contrasts between pre- and post-drug conditions (text S1) revealed the spatial location of the observed changes in connectivity.

### Structural connectivity

**Figure 7:**
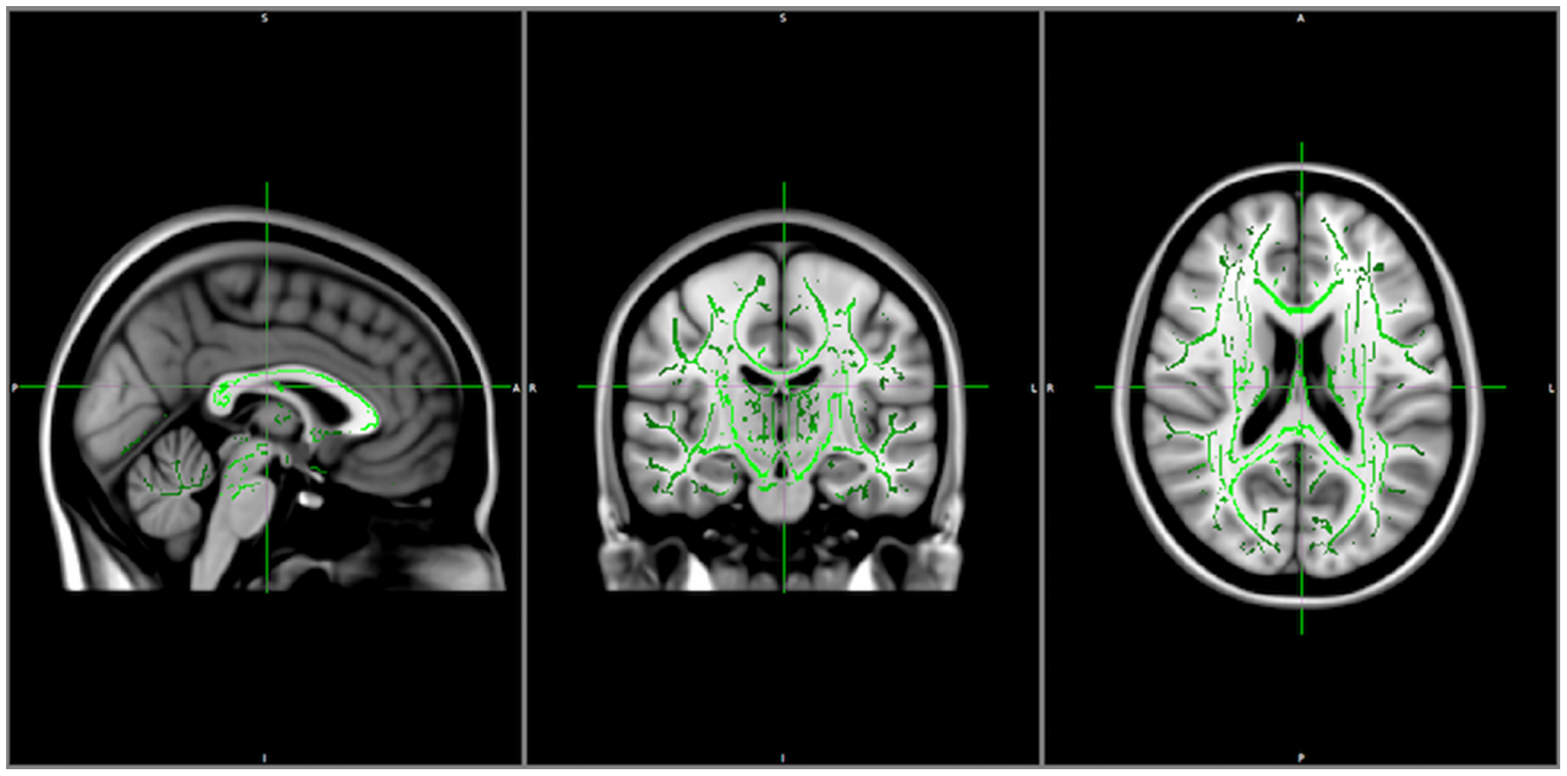
Structural connectivity analysis. TBSS analysis between the two groups (modafinil and placebo) shown on the MNI152 template. The FA skeleton (green) is used to extract data and compare areas of high anisotropy. No significant differences are observed between the two groups or between the pre-post drug/placebo conditions.

Computation of FA maps, combined with TBSS voxel wise analysis of multi-subjects diffusion data within the same group (modafinil and control) using time (i.e., before and after drug consumption) as covariate for the design matrix did not reveal differences in terms of structural connectivity of any study subject before and after placebo or modafinil administration (Figure 7).

## Discussion

Modafinil is today studied as potential cognitive enhancer [14], [15], [45], [46].

Our results indicate that a single dose of modafinil improves cognitive performance (Gf) and produces a statistically significant increased activation of FPC and DAN networks. These data intend to provide the starting point for further investigations aimed at assessing the potential implementation of the drug in pathophysiological conditions like brain aging and age-associated memory impairments.

Strong pre-clinical and clinical data support the use of modafinil. Modafinil administration has been shown to improve cognitive functions in mice [47], rats [48], sleep-deprived healthy adults [49], substance abusers [46], [50], and healthy adults [51], [14]. Cognitive improvement has been observed in several domains, including attentional control, working memory, and fluid reasoning, all processes that are critical components of Gf performance.

As mentioned above, modafinil mechanisms of action are complex and involve modulations of several neurotransmitter systems [52]. However, given the key role exerted by modafinil on dopamine, the drug appears to mainly promote enhanced learning by improving dopaminergic neurotransmission in the prefrontal cortex (PFC).

Modafinil administration is also associated with enhanced functional connectivity in the locus coeruleus (LC-PFC), a brain area, activated by norepinephrine, that critically regulates attention and high cognitive functions. Moreover, it should be noted that dopaminergic innervation from the ventral tegmental area and the LC [53] also modulates ACC activity.

A recent revision [54] of rs-fMRI BOLD imaging data across 19 independent studies (performed on almost one thousand subjects) suggests that the ACC is one of the most prominent functional hub in the brain. The ACC plays an important role in the regulation of attention, emotions, and reward-based decision processes [55].

Furthermore, combined findings in the field of neuroimaging and clinical neuropsychology have demonstrated an association between fluid reasoning, executive functions, working memory tasks and the neural activation of PFC-associated networks. These studies also underline the important role played in the process by the superior parietal temporal and occipital cortex as well as by subcortical regions and the striatum in particular [56].

A parieto-frontal integration theory of intelligence (P-FIT) has been recently proposed in a revision of thirty-seven structural and functional neuroimaging studies that investigated “intelligence and reasoning tests” [56]. According to the theory, Gf needs the activation of selective frontal and parietal brain regions along with specific temporal and occipital areas.

P-FIT supports the view that general intelligence is not localized or dependent on the activity of a specific anatomical region of the brain. The theory instead favors the notion that general intelligence involves the coordinated activation of a complex network that comprises multiple brain regions [56] like the Brodmann areas (BAs), the dorsolateral prefrontal cortex (BAs 6, 9, 10, 45, 46, 47), the inferior (BAs 39, 40) and superior (BA 7) parietal lobule, the ACC (BA 32), and regions within the temporal (BAs 21, 37) and occipital (BAs 18, 19) lobes [56], investigated in our study.

As far as modafinil Gf effects, regression analysis of APM results indicates that in the treated group, but not in the placebo cohort, there is an improvement in subject that are low performing at baseline when these individuals are challenged with items of medium levels of difficulty. This finding is in line with the idea that the drug can work better in individuals performing at submaximal levels [57]. This result is in accordance with investigation employing modafinil to compensate frank cognitive deficits in psychiatric patients [52]. Our regression analysis also shows that the drug can work to some extent but cannot increase performances when subjects are facing items of high levels of difficulty. Interestingly, the analysis also revealed a placebo effect in subjects starting with lower baseline scores and dealing with low level of difficulty items. This placebo effect is likely due to an overall increase in motivation. Of note, modafinil has no a similar effect in the same set of individuals (i.e.: subjects starting with lower baseline scores and dealing with low level of difficulty items). Effects of modafinil, observed only in low performing subjects, are in line with previous observations reporting no or even negative modafinil effects on cognition of healthy subjects [57]. The drug has indeed been reported to decrease performance in highly performing individuals, thereby indicating an inverted U-shaped dose-response relationship [57]–[60].

APM is probably modulated by training on working memory. Enhanced working memory has been associated with variations in PFC activity [61]. Interestingly, training tasks that are aimed at improving working memory and Gf also promote increased D1 receptor density in the PFC throughout inhibition of DAT1 activity [62]. These results suggest an important role for dopaminergic neurotransmission in these processes and provide a neurobiological substrate for the modafinil effects.

It should also be emphasized that, despite the common assumption that DAT1 is strictly localized in the striatum (and absent in the frontal cortex), data on rodents demonstrate significant levels of transporter binding in the ACC, prelimbic, and rostral areas of the frontal cortex [63] while extrastriatal DAT1 localizations have been confirmed in post-mortem human brains where the transporter has been found in the neocortex although at lower density when compared its striatal distribution [64].

Our rs-fMRI findings suggest that modafinil modulates functional connectivity in specific RSNs like the lFPC, DAN, and bilateral EsV. In agreement with previous data showing effects on the PFC [1], we have found that the drug significantly enhanced ACC functional connectivity within the IFPC. An interesting study [65] underlines the fact that the brain is organized in networks that are divided in subnetworks. These subnetworks are associated with different cerebral functions. The FPC network is constituted by two different subnetworks that are strongly lateralized. The right FPC (insular areas) supports perception–somesthesis–pain domains. The lFPC (Broca’s and Wernicke’s areas) strongly correlates with cognition–language domains. A recent study indicated neural substrates of Gf and showed that the process is modulated by the lFPC [66]. These left specializations offer a potential neural substrate for the modafinil- induced lateralized effects that we observe on the lFPC. The ACC result is in line with the increased ACC functional connectivity promoted by modafinil in a population of metamphetamine addicts who performed deterministic and associative learning tasks during fMRI [15].

EsV is known to play an important role in attention as selection of relevant information is mediated by visual attention [67] and several studies indicate that, at the neural level, the act of directing attention to a particular stimulus is often associated with increased EsV activation [33].

Our findings are not in line with a recent fMRI study in which modafinil (100 mg) was administered for seven days and showed no effect on attentional tasks while promoted decreased ACC connectivity [68]. The discrepancy between the two set of findings may be due to differences in drug regimens (acute versus chronic) as chronic exposure to the drug likely favours different re-arrangements of brain regions in response to receptor desensitization.

Attention processes involve the activation of DMN, FPC and DAN. DAN is hypothesized to modulate externally directed attention by amplifying or attenuating the saliency of relevant and irrelevant cues [33]. Pharmacological modulation of dopaminergic neurotransmission strongly affects attention and DAN activity. In that respect, methylphenidate is known to block dopamine reuptake and increase DAN activation upon visual attention and memory tasks [69]. Modafinil also blocks DAT1, thereby providing a common mechanism of the action on DAN activity that we observed.

A recent study, investigated effects of modafinil on DMN activity in healthy subjects [45]. In this study, subjects performed a simple visual sensorymotor task upon slow event-related fMRI and the authors found that modafinil promoted DMN deactivation as well as faster reaction time. When considering the study experimental design several differences emerge in comparison to our work [different modafinil doses, wider age range of study subjects as well as the type of task chosen during fMRI (visual sensorimotor)]. These differences can, at least in part, help to explain the discrepancy with our findings as we do not observe changes in DMN activity. However, modafinil appears to induce a plastic reorganization of specific brain regions involved in learning and Gf.

## Conclusions

Our results suggest that modafinil positively modifies brain connectivity with a pattern that can be related to Gf. Changes of functional connectivity induced by modafinil suggest a speculative hypothesis by which Gf modulation is associated with a concerted enhanced activity of visual-spatial, memory, and attentive skills.

A word of caution should be spent on the general use of modafinil. Modafinil has been originally indicated as cognitive enhancer with low risk of inducing addiction and few side effects. However, it is becoming clearer that the drug is acting on dopaminergic neurotransmission and therefore, as other classic psychostimulants, poses addiction risks. Furthermore, the long-term modafinil effects are still not completely explored. Better drugs with less addictive profiles will definitely provide more effective tools to safely address the issue of pharmacological modulation of cognition in physiology and pathology.

## Supporting Information

Text S1 **Rs-fMRI acquisition and statistical analysis.**
(DOC)

Protocol S1 **Trial protocol.**
(PDF)

Checklist S1 **CONSORT Checklist.**
(PDF)
