# Supplementary material for: Acute Effects of Modafinil on Brain Resting State Networks in Young Healthy Subjects
Source: PLoS One. 2013 Jul 25;8(7):e69224. doi: 10.1371/journal.pone.0069224 (PMC3723829; doi:10.1371/journal.pone.0069224)
Supplement: Text S1 — Rs-fMRI acquisition and statistical analysis. (DOC) [file pone.0069224.s001.doc]

**Supporting Information: Text S1**

***Rs-fMRI acquisition and statistical analysis***

Functional image time-series were first corrected for differences in slice acquisition times, de-trended using a temporal high-pass filter with cut-off set to 2 cycles per time-course and motion-corrected. Motion parameters were stored in log files and inspected to check that no significant differences in motion were present between runs. Pre-processed functional images were then realigned with the structural images and warped into standard Talairach anatomical space using a 12-parameter affine transformation as previously described [1]. Upon this normalization procedure,functional time series were resampled at a voxel size of 3 mm × 3 mm × 3 mm.

RSNs were investigated by means of independent component analysis (ICA) [2]. First, single subject ICA was performed separately for each of the three runs using a plugin extension of BrainVoyager QX based on the FastICA algorithm [3]. Thirty ICs were extracted for each data set and scaled to spatial z-score maps. In each IC map, the z-score value associated to a given voxel reflects the weight of IC time course with respect to the relative measured BOLD data, thereby providing an indirect indication of functional connectivity [2]. The number of ICs to be extracted is a free parameter of the analysis and has been chosen on the basis of previous works [4]. ICA decompositions from three within-subject repetitions per session were clustered using a self-organizing group-level ICA (sog-ICA) algorithm [5] implemented in BrainVoyager QX. The algorithm uses a linear correlation coefficient as the similarity measure between spatial maps and based on a hierarchical clustering procedure. For each subject, the procedure yielded 30 clusters composed of 4 components each. The 4 components were averaged in order to obtain a set of 30 within-subject components for session. The individual clustered components from all subjects and sessions were entered in a second sog-ICA analysis in order to obtain group level ICs. This analysis yielded 30 group level ICs, each one deriving from the clustering of 52 individual components. We chose to derive group level ICs including both pre and post placebo/drug sessions in order to avoid any bias toward a specific experimental condition, in the following analysis aimed at comparing changes in functional connectivity due to the different treatments. The 52 individual components of each group IC were used to compute a corresponding voxel-wise group random-effects t-map. We obtained 30 group maps that were visually inspected to recognize the most physiologically relevant and consistently reported RSNs [1], [6]. Further analysis was carried out only on selected RSNs. The group map of each network was threshold at a significance level of p=0.05 (Bonferroni corrected for multiple comparisons). This threshold map was then used to create a mask of voxels representing the whole network. Distinct masks representing different nodes of the RSNs were also obtained considering the 200 most significant voxels around each local Z-score maximum. For each mask, we then extracted the 52 Z-score values (from the 52 single-subject ICs forming that group RSN) representing individual levels of connectivity in the whole network and in single nodes during the different experimental conditions. To avoid ICA sign ambiguity, each component sign was adjusted in such a way to have all Z-scores positive [7]. Individual Z-scores in each node were compared by means of a mixed design ANOVA with the factors group (drug, placebo) and time (pre, post) in order to evaluate statistically significant connectivity changes due to treatment (modafinil or placebo). ANOVAs were followed by Duncan’s post-hoc tests with Bonferroni correction for multiple comparisons. To avoid false positives, a Bonferroni correction with a number of nodes =22 (see results) was performed.

Group-level t-maps resulting from direct voxel-by-voxel contrasts between pre and post-drug conditions were also produced. These maps were threshold at p=0.05, corrected for multiple comparisons using a cluster size algorithm [8] based on Monte Carlo simulations implemented in BrainVoyager QX. A threshold of p=0.005 at the voxel level and an estimate of the spatial correlation of voxels were used as input in the simulations, yielding a minimum cluster size of seven voxels to obtain a p=0.05 corrected for multiple comparisons.

**References**

1. Mantini D, Perrucci MG, Del Gratta C, Romani GL, Corbetta M. (2007) Electrophysiological signatures of resting state networks in the human brain. Proc NatlAcadSci U S A. 104:13170-13175.

2. McKeown MJ, Makeig S, Brown GG, Jung TP, Kindermann SS, et al. (1998) Analysis of fMRI data by blind separation into independent spatial components.Hum Brain Mapp. 6(3):160-88.

3. Hyvarinen A. (1999) Fast and robust fixed-point algorithms for independent component analysis. IEEE Trans Neural Netw.10(3):626-34.

4. Greicius MD, Flores BH, Menon V, Glover GH, Solvason HB, et al. (2007) Resting-state functional connectivity in major depression: abnormally increased contributions from subgenual cingulate cortex and thalamus. Biol Psychiatry. 62(5):429-37.

5. Esposito F, Scarabino T, Hyvarinen A, Himberg J, Formisano E, et al. (2005) Independent component analysis of fMRI group studies by self-organizing clustering. Neuroimage.25(1):193-205.

6. Van den Heuvel MP, Hulshoff Pol HE. (2010)Exploring the brain network: a review on resting-state fMRI functional connectivity. Eur Neuropsychopharmacol. 20(8):519-34.

7. Esposito F, Pignataro G, Di Renzo G, Spinali A, Paccone A, et al. (2010) Alcohol increases spontaneous BOLD signal fluctuations in the visual network. Neuroimage.53(2):534-43.

8. Forman SD, CohenJD, Fitzgerald M, Eddy WF, Mintun MA, et al. (1995) Improved assessment of significant activation in functional magnetic resonance imaging (fMRI): use of a cluster-size threshold. MagnReson Med. 33(5):636-47.
