# Supplementary material for: Acute Effects of Modafinil on Brain Resting State Networks in Young Healthy Subjects
Source: PLoS One. 2013 Jul 25;8(7):e69224. doi: 10.1371/journal.pone.0069224 (PMC3723829; doi:10.1371/journal.pone.0069224)
Supplement: Protocol S1 — Trial protocol. (PDF) [file pone.0069224.s002.pdf]

UNIVERSITA' G. D'ANNUNZIO
ASL CHIETI
COMITATO DI ETICA
PER LA RICERCA BIOMEDICA
RICEVUTO IN DATA 14/10/2009

PROTOCOLLO DELLO STUDIO SPONTANEO

TITOLO DELLO STUDIO: Valutazione dell'effetto della somministrazione di Modafinil 100mg sulla connettività cerebrale, mediante tecniche fRMN ed EEG.
Codice identificativo: Modrest

Responsabile scientifico: Dott. Franco Cilli
Tutor: Prof. Stefano Sensi
Co-sperimentatori: Dott.ssa. Francesca Sensi, Ing. Dante Mantini, Dott. Massimo Caulo, Dott. Roberto Esposito.

RIASSUNTO DELLO STUDIO

Lo studio prevede il reclutamento di quattro gruppi di soggetti, il primo di età compresa dai 65 agli 75 anni, il secondo dai 25 ai 35 anni, e relativi gruppi di controllo. I primi due gruppi verranno sottoposti a fRMN e contestualmente ad esame EEG, sia prima che dopo l'assunzione di un farmaco, il Modafinil, utilizzato come indicazione esclusiva nella narcolessia e considerato un enhancer delle funzioni cognitive. Le misurazioni con fRMN saranno eseguite nello stato di riposo (Resting State), uno stato che viene ritenuto di estremo interesse nella valutazione della funzionalità cerebrale in rapporto alle diverse aree che durante questa condizione si attivano o si deattivano. Il farmaco verrà somministrato in dose singola e le misurazioni con fRMN verranno eseguite secondo una tempistica che sarà posta in relazione alla farmacocinetica del composto.

INTRODUZIONE E RAZIONALE

Lo studio proposto ha come punto di partenza la modificazioni del Resting State associate a particolari stati mentali e processi cognitivi.

Resting State e connettività cerebrale.

Negli ultimi venti anni gli studi funzionale del cervello hanno avuto un largo sviluppo, grazie all'impiego di tecniche di indagine sempre più avanzate (elettroencefalografia, magnetoencefalografia e a partire dagli anni '90 anche la tomografia ad emissione di positroni e la risonanza nucleare magnetica.
Nell'ultima decade si è affermata tra i ricercatori l'ipotesi che a riposo il cervello sia impegnato in un'attività spontanea, definita in letteratura come default mode of brain function, baseline state, o anche conscious resting state .
Le aree cerebrali coinvolte in tale attività di fondo costituirebbero una rete funzionale, detta default mode network (DMN).
Per rete viene qui intesa l'attività spontanea simultanea di aree anatomicamente segregate, in accordo con il concetto di connettività funzionale: regioni che si attivano in maniera concorde appartengono ad una rete funzionale, cioè concorrono a realizzare una stessa funzione.
La default mode network è costituita dalle aree cerebrali la cui attività aumenta durante lo svolgimento di compiti cognitivi semplici oppure a riposo rispetto all'esecuzione di task più complessi.
Le regioni attribuite alla DMN da studi di risonanza magnetica funzionale corrispondono significativamente alle aree cerebrali metabolicamente più attive nello stato di riposo.

Le maggiori potenzialità dell'analisi dello stato di riposo risiedono nell'individuazione di alterazioni della connettività cerebrale associate a patologie neurologiche e psichiatriche: ciò potrebbe infatti contribuire alla caratterizzazione fisiopatologica e alla diagnosi preclinica di tali malattie.
Siamo in possesso a tutt'oggi di una quantità cospicua di dati che pongono in relazione le modificazioni strutturali e funzionali associate a patologie neurodegenerative cerebrali e modificazioni della connettività cerebrale che consistono in una minore o maggiore connessione di determinate aree cerebrali, in condizioni di resting state. Nel soggetto sano in condizione di riposo, come abbiamo visto alcune aree si attivano considerevolmente e si deattivano nel momento in cui il soggetto viene sottoposto a stimolo cognitivi. In soggetti con patologie neurodegenerative come l'Alzheimer, l'entità della connessione di determinate aree cerebrali è considerevolmente minore che nel sano. In pratica ciò equivale a dire che maggiore è la degenerazione neuronale, minore è la capacità nei soggetti esaminati di passare dallo stato di riposo ad uno stato di attività cerebrale che caratterizza l'esecuzione di compiti cognitvi di diverso grado di complessità. Il malato in sintesi resta "attivo" nel suo stato di riposo più del sano. Tracciando una linea ideale che unisce due opposte polarità possiamo rappresentare i due poli contrapposti come insieme composto da soggetti completamente sani da una parte e soggetti malati dall'altra. Nel mezzo ci sono condizioni che degradano progressivamente dal soggetto giovane sano fino all'Alzheimer conclamato, passando per l'anziano sano e per gli stati precoci dell'Alzheimer.
Nello studio in oggetto, ci riproponiamo di mettere in evidenza la capacità del Modafinil, un farmaco che promuove la veglia di diverse specie compreso l'uomo, nel modificare lo stato di riposo nei soggetti sottoposti a trattamento, causando una maggiore connettività cerebrale di quest'ultimo. Il nostro intendimento è di valutare secondo modelli statistici l'entità della modificazione dello stato di riposo nei soggetti in esame e rimarcare eventuali differenze quali-quantitative fra i due gruppi. Un esito statisticamente significativo, relativamente alla capacità del farmaco di favorire una maggiore connettività nei soggetti dei due gruppi, sarebbe un indicatore predittivo della capacità del farmaco stesso di potenziare le capacità cognitive. Allo stesso tempo verrà valutata la differenza fra il gruppo "giovani" e il gruppo "anziani", quale modalità di risposta specifica nel soggetto anziano rispetto al soggetto giovane.

Farmacologia del Modafinil: indicazioni terapeutiche, meccanismo d'azione, farmacocinetica, effetti collaterali

Il Modafinil (commercializzato col nome di Provigil) è un farmaco stimolante prodotto dalla Cephalon, ed è approvato dal S.S.N. prevalentemente per il trattamento della narcolessia ed i disturbi ad essa correlati. Il Modafinil, come per altri stimolanti, aumenta il rilascio di molti neurotrasmettitori specialmente monoamminici ma innalza anche i livelli di istamina nell'ipotalamo, cosa che ha indotto alcuni ricercatori a considerare il Modafinil come un "agente promotore della veglia" piuttosto che uno stimolante assimilabile alle anfetamine (come dalle differenze dimostrate sulla distribuzione dei geni proto-oncogeni C-Fos indotta dal modafinil comparato alle anfetamine). Il modafinil è anche indicato, sebbene non approvato, nel trattamento della Sindrome da deficit di attenzione e iperattività o ADHD, , della depressione, Sindrome da astinenza da cocaina, Morbo di Parkinson, schizofrenia, e disturbi relativi all'affaticamento. Negli Stati Uniti tuttavia, per legge, comunque, Cephalon non è autorizzata alla vendita del Modafinil per condizioni non ufficialmente approvate dall'FDA.

Il Modafinil e il suo precursore chimico, l'Adrafinil furono sviluppati nei Lafon Laboratories, una società francese acquisita dalla Cephalon nel 2001. Il Modafinil è il metabolita primario dell'adrafinil e, sebbene la loro azione sia molto simile, l'adrafinil richiede un dosaggio maggiore per ottenere effetti della stessa entità.

Allo stato attuale il Modafinil è stato approvato in Italia con la sola indicazione della narcolessia e dei disturbi ad essa correlati.

Utilizzo off label

Il Modafinil è largamente utilizzato per sopprimere il bisogno del sonno. E' anche utilizzato per trattare l'affaticamento non correlato alla mancanza di sonno, nel trattamento dell'ADHD e come coadiuvante agli antidepressivi (particolarmente negli individui con notevole affaticamento residuo). C'è un acceso dibattito nel quale viene discusso se gli effetti del Modafinil mostrati in persone sane e con sonno regolare sono sufficienti per considerarlo un "potenziatore cognitivo"." I ricercatori concordano che il Modafinil potenzia alcuni aspetti della memoria, come il digit span, manipolazione digitale e nella memoria atta al riconoscimento di forme, oggetti, volti, ecc.., ma i risultati relativi alla memoria spaziale, alle funzioni esecutive e all'attenzione sono dubbi. Alcuni degli effetti positivi del Modafinil potrebbero essere limitati a individui "poco performanti" o persone con un quoziente d'intelligenza basso. C'è anche la prova che esso ha effetti neuroprotettivi Modafinil potrebbe anche essere un efficace e ben tollerato trattamento nei pazienti con disordine affettivo stagionale o depressione invernale.

Doping

Il Modafinil in passato è stato usato come agente dopante da molti atleti. C'è una prova "aneddotica" che testimonia che il modafinil incide sulle prestazioni fisiche degli atleti. Nel 2004 il Modafinil è stato aggiunto nella "Lista Proibita" della Worl Anti-Doping Agency come stimolante proibito.

Sclerosi Multipla

Il Modafinil è stato usato per alleviare i sintomi della fatica neurologica riportata da alcuni pazienti affetti da sclerosi multipla.

ADHD o sindrome da deficit di attenzione e iperattività

Sono presenti sette studi in letteratura si sperimentazioni cliniche che indicano l'uso del Modafinil per il trattamento dell'ADHD

Usi sperimentali

Dipendenza da cocaina
Perdita di peso
Cirrosi biliare primitiva
Danni cognitivi post-chemioterapia
Elevazione del tono dell'umore

Controindicazioni ed avvertenze

Dati della letteratura suggeriscono cautela prima di iniziare un trattamento con Modafinil, in particolare per quei pazienti che hanno mostrato ipersensibilità al farmaco o ai costituenti dello stesso, in pazienti con problematiche cardiovascolari in special

modo in relazione all'uso di altri stimolanti, condizioni cardiache particolari come ipertrofia ventricolare sinistra, prolasso della valvola mitrale.
Sebbene non sia stato discusso in letteratura, gli addensanti standard utilizzati per il Modafinil contengono farina di grano, pertanto soggetti con celiachia dovrebbero evitare l'uso di tale farmaco.

Reazioni avverse gravi

Il Modafinil può indurre reazioni dermatologiche severe che richiedono l'ospedalizzazione. Dalla data di inizio della commercializzazione, nel dicembre 1998, al Gennaio 2007, l'FDA ha ricevuto sei casi di reazioni cutanee avverse gravi associate al modafinil, riguardanti soggetrti adulti ed in età pediatrica, che includevano: l'eritema multiforme (EM), la sindrome di Stevens-Jhonson, la necrolisi epidermoidale tossica, rash con eosinofilia e sintomi sistemici.
L'avviso emanato dalla FDA assume un notevole rilievo. Nello stesso avviso, l'FDA ha messo in evidenza anche un angioedema e una reazione da ipersensibilità multi organo, negli studi post-marketing.
I pazienti con ansia severa dovrebbero essere attentamente controllati, poiché il modafinil può esacerbare tale condizione. Potrebbe essere necessario la cosomministrazione di ansioltici. La pressione sanguigna deve essere stabilizzata prima di iniziare il trattamento.
I pazienti dovrebbero informare il proprio medico curante in merito ad ogni eventuale trattamento a cui sono sottoposti, dato che il modafinile può interagire con un gran numero di farmaci.
Si conosce relativamente poco in merito alla sicurezza durante la gravidanza o l'allattamento . Studi su coniglie gravide, suggeriscono l'eventualità di una maggiore probabilità di difetti alla nascita. Non ci sono sudi adeguati e ben controllati con Modafinil in donne gravide. Il Modafinil dovrebbe essere usato in gravidanza solo se il potenziale beneficio della madre supera il potenziale rischio del feto.
Non è noto se il Modafinil vine escreto nel latte. Il Modafinil riduce l'efficacia dei contraccettivi. Possibilmente alcol ed altre sostanze ad azione depressiva per il SNC, dovrebbero essere evitati durante il trattamento.

Effetti Collaterali

Gli effetti collaterali più comuni, quando il farmaco è assunto nelle dosi prescritte secondo le indicazioni riconosciute del farmaco, sono i seguenti:

Effetti Comuni

Cefalea (34 vs 23%)
Nausea (11 vs 3%)
Non comuni
Nervosismo
Insonnia
Ansia
Anoressia
Bocca secca
Rari
Dolori al petto
Ipertensione

Tachicardia
Vasodilatazione
Vertigini
Parestesie
Faringite
Sono stati riscontrati in aggiunta, effetti gastrointestinali, che possono essere alleviati assumendo il farmaco a stomaco pieno. Sono stati riportati anche irritazioni della pelle ed aggressività, sebbene moto raramente.
La maggioranza degli effetti recedono dopo alcune settimana senza bisogno di ridurre le dosi. Solo la cefalea e l'ansia sono risultate commisurate al dosaggio ed hanno beneficiato di una riduzione della dose o da somministrazioni a dosi refratte e a dosaggi minori.
In un singolo caso una extrasistole ventricolare è apparsa correlata casualmente alla somministrazione di Modafinil.
Il Modafinil interagisce con i contraccettivi orali e l'effetto di questa interazione dura all'incirca un mese.
La tossicità del Modafinil ha un'ampia variabilità di specie. Nei topi e nei gatti, la dose media letale LD50, è approssimativamente o leggermente superiore a 1250 mg/kg. I valori di range, in seguito a somministrazione orale di LD50, riportati nei ratti variano da 1000mg/kg a 3400mg/kg. La LD50 endovenosa per i cani si aggira sui 300mg/kg. In clinical trials effettuati su soggetti umani che assumevano dosi di 1200 mg di farmaco giornaliero per 7-21 giorni, o che avevano assunto una dose singola di 4500 mg, non ha causato effetti pericolosi per la vita, sebbene siano stati osservati un certo numero di esperienze avverse quali agitazione/eccitazione, insonnia, ansia, irritabilità, aggressività, confusione, tremori palpitazioni, disturbi del sonno, nausea e diarrea. Nel 2004 la FDA non aveva una cognizione esatta della overdose fatale di Modafinil assunto da solo (in opposizione a una somministrazione multipla di farmaci includenti anche il Modafinil). Di conseguenza la LD50 orale del Modafinil non è conosciuta in maniera esatta. Sembra comunque che la LD50 orale del Modafinil sia molto superiore a quello della caffeina.

Farmacologia
L'esatto meccanismo d'azione del Modafinil non è chiaro, sebbene numerosi studi in vitro abbiano mostrato, che esso aumenti i livelli di monoamine, come la dopamina nello striato e nel nucleo accumbens[x], la noradrenalina nell'ipotalamo e nel nucleo preottico[x] e la serotonina nell'amigdala e nella corteccia frontale. Se è risaputo che la co-somministrazione di antagonisti della dopamina, diminuisce gli effetti stimolanti della dopamina, gli stessi non inibiscono l'effetto di promozione della veglia del Modafinil. Il Modafinil attiva il circuito glutaminergico inibendo allo stesso tempo la trasmissione GABA-ergica. Si ritiene che il Modafinil abbia meno potenziali effetti di abuso degli altri stimolanti a causa dell'assenza di significativi effetti euforizzanti o piacevoli.
Gli effetti stimolanti centrali del Modafinil mostrano aspetti correlati alla dose e tempo. Gli effetti tendono ad essere potenziati dalla clorazione , ma ridotti dalla metilazione. Il Modafinil blocca il reuptake della della norepinefrina dai terminali noradrenergici dei neuroni che promuovono il sonno, dei nuclei preottici ventrolaterali. Tale meccanismo potrebbe parzialmente responsabile per l'effetto di promozione della veglia. Il Modafinil ha un coefficiente di binding (Ki) di circa 4,000 nmol/L per il trasportatori del reuptake della dopamina, e oltre i 10.000 nmol/L per i trasportatori della norepinefrina.
Un nuovo meccanismo d'azione prposto coinvolge un peptide cerebrale chiamato orexina, conosciuto anche come ipocretina. I neuroni contenenti orexina sono stai trovati nell'ipotalamo , ma proiettano in differenti parti del cervello, incluse molte aree

che regolano la veglia. L'attivazione di questi neuroni aumentano la dopamina e la norepinefrina in queste aree, e stimolano i neuroni istaminergici tubero mammillari che aumentano i livelli di istamina nelle stesse aree.
Ci sono due tipi di recettori per l'ipocretina, chimati hcrt1 e hcrt2. Studi sugli animali hanno mostrato che animali con deficit del sistema dell'orexina mostrano segni e sintomi simili alla narcolessia.

Farmacocinetica
Il Modafinil induce gli enzimi citocromo 450 CYP1A2, CUP1A2 e CYP3A4, e inibisce i CYP2C9 e CYP2C19 in vitro. Può anche indurre la P-glicoproteina, con effetto su quei farmaci trasportati dalla Pgp, come la digossina.
La biodisponibilità del Modafinil è superiore all'80% della dose somministrata. In vitro le misurazioni indicano che il Modafinil è legato al 60 % alle proteine del plasma alla concentrazione clinica del farmaco. Questa concentrazione in realtà varia molto poco in relazione alla dose somministrata.
La Cmax si rileva approssimativamente 2-3 ore dopo la somministrazione. Il cibo rallenta al'assorbimento, ma non influisce sull'assorbimento totale. L'emivita è generalmente di 10-12 ore. Soggetta a differenze rispetto ai genotipi CYP, alla funzionalità epatica e renale. E' metabolizzato nel fegato, e il suo metabolita inattivo è secreto nella urine.

OBIETTIVI

Ci si propone di utilizzare il potenziale delle tecniche fRMN ed EEG nel rivelare il comportamento organizzato delle aree corticali appartenenti alle diverse reti cerebrali. Se l'attività cerebrale nello stato di riposo è stata studiata diffusamente negli ultimi anni, non risulta ancora chiaro quali siano i meccanismi alla base dei processi di connettività funzionale. A questo scopo, studieremo l'effetto della somministrazione di un farmaco come il Modafenil, a cui è attribuito un effetto di potenziamento delle capacità cognitive, sull'organizzazione cerebrale nello stato di riposo.
Gli obiettivi dello studio saranno i seguenti:
1) Verificare se c'è un globale aumento nella connettività cerebrale nello stato di riposo, sia nell'anziano che nel giovane, in relazione alla somministrazione del farmaco.
2) Ottenere mappe spaziali di connettività, prima e dopo la somministrazione del farmaco, e stabilire se le eventuali alterazioni n sono localizzate in specifiche aree cerebrali.
3) Mettere in relazione le aree in cui l'azione del farmaco è maggiore con le aree cerebrali con ridotta funzionalità nelle varie patologie per cui il farmaco è tipicamente impiegato.
4) Valutare le differenze in termini di connettività cerebrale fra il gruppo di soggetti giovani e quello dei soggetti anziani, quale misura indiretta di una diversa efficacia in differenti gruppi di età.

RISULTATI ATTESI
Lo studio permetterà di identificare le aree cerebrali su cui il farmaco agisce in termini di aumentata connettività cerebrale, misurata in termini di correlazione fra i time-course del segnale fRMN. Sulla base di precedenti studi di neuroimaging, si prevede di ottenere un significativo aumento con la somministrazione del Modafinil, sia nei giovani che negli anziani, nella corteccia mediale prefrontale. Inoltre, gli effetti comportamentali legati al farmaco riportati in letteratura suggeriscono che il Modafinil possa indurre una

aumento globale (non specifico per una determinata area) significativamente maggiore negli anziani che nei giovani. Questo effetto dovrebbe essenzialmente tendere a ripristinare una ridotta connettività funzionale, riportata per la popolazione anziana rispetto alla popolazione giovane. Fra prima e dopo la somministrazione del farmaco, ci attendiamo di riscontrare una ridotta efficacia nei giovani rispetto agli anziani. Le eventuali differenze potranno essere localizzate nelle aree parietali ed occipitali, su cui si ritiene che il farmaco abbia un effetto ridotto.

DISEGNO DELLO STUDIO

Verranno selezionati 2 gruppi di 20 soggetti ciascuno, da sottoporre a somministrazione di dose singola di farmaco (Modafinil 100mg), e relativi gruppi di controllo.

I gruppi a cui verrà somministrato il farmaco saranno composti da soggetti giovani in un gruppo, di età compresa tra i 25 e i 35 anni, e soggetti appartenenti ad una fascia di età compresa tra i 65 e gli 75 anni nell'altro gruppo. I gruppi di controllo avranno la stessa consistenza numerica e dovranno corrispondere per età e sesso ai gruppi sottoposti a trattamento.

Tutti i partecipanti allo studio saranno sottoposti a misurazioni fRMN ed EEG in condizioni di Resting State, sia prima che dopo la somministrazione del farmaco. La seconda misurazione avverrà circa tre ore dopo la somminisrazione dello stesso farmaco per considerazioni inerenti la farmacocinetica del composto. In questo modo sarà possibile valutare le differenze nella funzionalità cerebrale in seguito all'effetto del Modafinil su determinate aree cerebrali.

La durata dello studio è circoscritta al tempo di esecuzione degli esami previsti e al processamento dei dati risultanti, con successiva analisi statistica degli stessi.

Popolazione

I soggetti da esaminare verranno selezionati fra gli iscritti di istituti quali l'Università delle Terza Età e fra gli studenti universitari.

I partecipanti allo studio dovranno essere esenti da malattie croniche invalidanti, da cardiopatie, insufficienza epatica e renale e malattie neurodegenerative quali l'Alzheimer, il M. di Parkinson ecc, dove l'evidente compromissione dell'integrità, della plasticità cerebrale e delle funzioni cognitive, interferirebbe significativamente con i risultati dello studio. Verranno esclusi anche soggetti con anamensi positiva di epilessia. Inoltre, allo scopo di eliminare l'interferenza dovuta all'interazioni fra i farmaci assunti ed il Modafinil, i soggetti sottoposti a farmaco dovranno essere drug free.

Verranno effettuati preventivamente test di personalità e neuropsicologici, allo scopo di escludere pazienti con gravi deficit cognitivi e malattie psichiatriche.La valutazione clinica dei partecipanti allo studio verrà fatta da medici e psicologi cosperimentatori in collaborazione con i medici curanti dei volontari partecipanti. A questo proposito a ciascun Curante verrà inviata una lettera formale indicante le finalità dello studio ed i criteri di inclusione nello stesso.

Tenuto conto della cinetica del farmaco (Cmax, emivita), la valutazione dello stato di riposo, verrà effettuata tre ore dopo l'assunzione del farmaco. Il farmaco verrà somministrato a stomaco vuoto, nella forma di compresse da 100mg (una singola compressa).

L'assegnazione dei soggetti a vari gruppi, una volta raggiunto il numero necessario di soggetti corrispondenti alle caratteristiche dello studio, verrà operata attraverso randomizzazione.

Considerazioni statistiche
In considerazione del fatto che il confronto dell'effetto della somministrazione del Modafinil viene fatto su più di due gruppi, verrà effettuata un'analisi della varianza (ANOVA). Questa è una tecnica statistica nata nell'ambito della tecnica sperimentale per valutare l'effetto di determinati fattori, variabili indipendenti – di tipo continuo o categoriale – sulla variabile dipendente di tipo continuo. Applicata all'analisi dei dati fRMN e EEG, l'ANOVA permetterà di identificare aree cerebrali che presentano un significativo aumento/diminuzione della connettività cerebrale, legata alla somministrazione del farmaco e all'età dei partecipanti alla sperimentazione. Tali alterazioni verranno considerate significative qualora il test relativo all'ANOVA dovesse fornire una probabilità inferiore alla soglia di $p=0.05$. In aggiunta all'ANOVA, potrà essere effettuato il confronto diretto dei risultati attenuti in due gruppi scelti, come ad esempio nei soggetti giovani prima e dopo la somministrazione del farmaco. In questo caso, verrà utilizzata un test t di Student, la cui soglia di significatività verrà fissata a $p=0.01$, eventualmente corretto per confronti multipli.

Trattandosi di studio a carattere esplorativo non si e' proceduto ad un dimensionamento campionario. Tuttavia si ritiene che un numero di soggetti pari a 15 per gruppo sia sufficiente ai fini della generazione di ipotesi da testare in un secondo tempo con studi mirati.

Raccolta e trasmissione degli eventi avversi gravi
Tutti gli eventi avversi gravi che si verifichino in seguito a somministrazione del farmaco e per i 3 giorni successivi verranno raccolti e trasmessi utilizzando la Scheda di segnalazione eventi avversi gravi (SEAG).
Indipendentemente dalla eventuale segnalazione mediante SEAG, tutti gli eventi avversi considerati correlati all'uso dei farmaci in studio e che configurino una forma di tossicità dovranno essere riportati nelle schede di raccolta dei dati sulla tossicità dei trattamenti.

Codifica del nesso causale tra trattamento ed evento inesistente:
improbabile: l'evento avverso è dovuto ad una causa alternativa più probabile (i.e. farmaci o malattie concomitanti), e/o la relazione temporale suggerisce come improbabile la relazione causale.

possibile: l'evento avverso potrebbe dipendere dall'uso del farmaco, non essendoci evidenze conclusive di cause alternative, ed essendo plausibile la relazione temporale, una relazione causale non può essere esclusa.

probabile: l'evento avverso potrebbe dipendere dall'uso del farmaco, essendo supportato dalla relazione temporale e non essendo verosimili altre cause.

certo: l'evento avverso è conosciuto come possibile reazione avversa del farmaco in studio e non ci sono ragionevoli spiegazioni alternative.

ASPETTI ETICI

Le procedure riportate nello studio riguardanti la conduzione, lo svolgimento e la documentazione saranno approntate tenendo fede fede ai principi etici riportati nella

Dichiarazione di Helsinki e sue revisioni. Lo studio verrà condotto tenendo conto dei requisiti regolatori e degli adempimenti di legge. In particolare, il riferimento normativo è rappresentato dal DL n.211, 24/06/2003 e DM 17/12/2004 sugli studi no profit.
Inoltre:
- Prima della registrazione, tutti i pazienti potenzialmente eleggibili riceveranno le informazioni complete sullo studio.
- Per poter essere registrati sarà richiesto ai pazienti il consenso al trattamento dei dati personali in forma anonima ed aggregata, ai sensi della legge 675/1996 e della legge legge 196/03 sulla tutela delle persone e rispetto al trattamento dei dati personali.
- I moduli di informazione dei pazienti e di esplicitazione del consenso sono riportati in allegato e una copia è allegata ad ogni scheda raccolta dati.

Garanzie oggettive

Le indagini si svolgeranno nei laboratori biomedici dell'I.T.A.B.
La strumentazione utilizzata è costituita dalla apparecchiatura per risonanza magnetica nucleare funzionale per immagini con campo magnetico 3 Tesla, dislocati presso l'ITAB e soddisfano le condizioni di sicurezza.
A tutti i soggetti verrà garantita la riservatezza dei dati trattati

BIBLIOGRAFIA

Resting State

1) Raichle ME, Snyder AZ. A default mode of brain function: a brief history of an evolving idea. Neuroimage. 2007 Oct 1;37(4):1073-82.

2) Raichle ME, MacLeod AM, Snyder AZ, Powers WJ, Gusnard DA, Shulman GL. A default mode of brain function. Proc Natl Acad Sci U S A. 2001 Jan 16;98(2):676-82.

3) Rombouts SA, Barkhof F, Goekoop R, Stam CJ, Scheltens P. Altered resting state networks in mild cognitive impairment and mild Alzheimer's disease: an fMRI study. Hum Brain Mapp. 2005 Dec;26(4):231-9.

4) Li SJ, Li Z, Wu G, Zhang MJ, Franczak M, Antuono PG. Alzheimer Disease: evaluation of a functional MR imaging index as a marker. Radiology. 2002 Oct;225(1):253-9.

Modafinil

1) Erman MK, Rosenberg R, For The U S Modafinil Shift Work Sleep Disorder Study Group. "Modafinil for excessive sleepiness associated with chronic shift work sleep disorder: effects on patient functioning and health-related quality of life." Primary Care Companion to the Journal of Clinical Psychiatry 2007;9(3):188-94. Full Text
2) "Provigil (Modafinil) Site." 12/24/1998 [1].
3) Ishizuka T, Murakami M, Yamatodani A (Jan 2008). Involvement of central histaminergic systems in modafinil-induced but not methylphenidate-induced increases

in locomotor activity in rats. Eur. J. Pharmacol. 578 (2-3): 209–15. DOI:10.1016/j.ejphar.2007.09.009.
4) Engber TM, Koury EJ, Dennis SA, Miller MS, Contreras PC, Bhat RV (Jan 1998). Differential patterns of regional c-Fos induction in the rat brain by amphetamine and the novel wakefulness-promoting agent modafinil. Neurosci. Lett. 241 (2-3): 95–8.
5) Biederman J, Pliszka SR (Mar 2008). Modafinil improves symptoms of attention-deficit/hyperactivity disorder across subtypes in children and adolescents. J. Pediatr. 152 (3): 394–9. DOI:10.1016/j.jpeds.2007.07.052.
6) Fava M, Thase ME, DeBattista C, Doghramji K, Arora S, Hughes RJ (2007). Modafinil augmentation of selective serotonin reuptake inhibitor therapy in MDD partial responders with persistent fatigue and sleepiness. Ann Clin Psychiatry 19 (3): 153–9. DOI:10.1080/10401230701464858.
7) Dackis CA, Kampman KM, Lynch KG, Pettinati HM, O'Brien CP (Jan 2005). A double-blind, placebo-controlled trial of modafinil for cocaine dependence. Neuropsychopharmacology 30 (1): 205–11. DOI:10.1038/sj.npp.1300600.
8) van Vliet SA, Vanwersch RA, Jongsma MJ, van der Gugten J, Olivier B, Philippens IH (Sep 2006). Neuroprotective effects of modafinil in a marmoset Parkinson model: behavioral and neurochemical aspects. Behav Pharmacol 17 (5-6): 453–62.
9) Turner DC, Clark L, Pomarol-Clotet E, McKenna P, Robbins TW, Sahakian BJ (Jul 2004). Modafinil improves cognition and attentional set shifting in patients with chronic schizophrenia. Neuropsychopharmacology 29 (7): 1363–73. DOI:10.1038/sj.npp.1300457.
10) Rammohan KW, Rosenberg JH, Lynn DJ, Blumenfeld AM, Pollak CP, Nagaraja HN (Feb 2002). Efficacy and safety of modafinil (Provigil) for the treatment of fatigue in multiple sclerosis: a two centre phase 2 study. J. Neurol. Neurosurg. Psychiatr. 72 (2): 179–83.
11) Rabkin JG, McElhiney MC, Rabkin R, Ferrando SJ (Dec 2004). Modafinil treatment for fatigue in HIV+ patients: a pilot study. J Clin Psychiatry 65 (12): 1688–95.
12) U.S. Food and Drug Administration. Prescription Drug Marketing Act of 1987 (PDMA), PL 100-293. Link
13) News Release (Cephalon). "Cephalon, Inc. Plans to Acquire France's Group Lafon." WEST CHESTER, Pa., Dec 3, 2001 (PR Newswire) Link
14) a b Turner DC, Robbins TW, Clark L, Aron AR, Dowson J, Sahakian BJ (2003). Cognitive enhancing effects of modafinil in healthy volunteers. Psychopharmacology (Berl.) 165 (3): 260–9. DOI:10.1007/s00213-002-1250-8.
15) a b Randall DC, Viswanath A, Bharania P, Elsabagh SM, Hartley DE, Shneerson JM, File SE (2005). Does modafinil enhance cognitive performance in young volunteers who are not sleep-deprived?. J Clin Psychopharmacol 25 (2): 175–9. DOI:10.1097/01.jcp.0000155816.21467.25.
16) a b Baranski JV, Pigeau R, Dinich P, Jacobs I (2004). Effects of modafinil on cognitive and meta-cognitive performance. Hum Psychopharmacol 19 (5): 323–32. DOI:10.1002/hup.596.
17) a b Müller U, Steffenhagen N, Regenthal R, Bublak P (2004). Effects of modafinil on working memory processes in humans. Psychopharmacology (Berl.) 177 (1-2): 161–9. DOI:10.1007/s00213-004-1926-3.
18) Randall DC, Shneerson JM, File SE (2005). Cognitive effects of modafinil in student volunteers may depend on IQ. Pharmacol. Biochem. Behav. 82 (1): 133–9. DOI:10.1016/j.pbb.2005.07.019.
19) Jenner, P (July 2000). Antiparkinsonian and neuroprotective effects of modafinil in the mptp-treated common marmoset. Experimental Brain Research 133: 178–188. DOI:10.1007/s002210000370.

20) Modafinil treatment in patients with seasonal affective disorder/winter depression: an open-label pilot study, Journal of affective disorders, 2004 Aug;81(2):173-8.
21) Rammohan, K W (2002). Efficacy and safety of modafinil (Provigil®) for the treatment of fatigue in multiple sclerosis: a two centre phase 2 study. Journal of Neurology Neurosurgery and Psychiatry 72: 179–183. DOI:10.1136/jnnp.72.2.179.
22) Lindsay SE, Gudelsky GA, Heaton PC (Oct 2006). Use of modafinil for the treatment of attention deficit/hyperactivity disorder. Ann Pharmacother 40 (10): 1829–33. DOI:10.1345/aph.1H024.
23) Psychopharmacologic Drugs Advisory Committee (transcript). FDA, 2006-03-23
24) Modafinil (CEP-1538) Tablets Supplemental NDA 20-717/S-019 ADHD Indication Briefing Document for Psychopharmacologic Drugs Advisory Committee Meeting (PDF). FDA, 2006-03-23. URL consultato il 2007-07-21.
25) Xiong, M.D., Glen L., Eric J. Christopher, M.D., and Jason Goebel, M.D. (2005). Modafinil as an Alternative to Methylphenidate as Augmentation for Depression Treatment. Psychosomatics 46: 578–579. DOI:10.1176/appi.psy.46.6.578.
27) Menza, MA (2000). Modafinil augmentation of antidepressant treatment in depression. J Clin Psychiatry 61: 378–381.
28) DeBattista, C (2004). A prospective trial of modafinil as an adjunctive treatment of major depression. J Clin Psychopharmacol 24: 87–90. DOI:10.1097/01.jcp.0000104910.75206.b9.
29) MacDonald, JR (2002). Modafinil reduces excessive somnolence and enhances mood in patients with myotonic dystrophy. Neurology 59: 1876–1880.
30) Webster, L (June 2003). Modafinil treatment of opioid-induced sedation. Pain Med 4: 135–140. DOI:10.1046/j.1526-4637.2003.03014.x.
31) Hurst, DL (March 2002). Use of modafinil in cerebral palsy. J Child Neurology 17: 169–172. DOI:10.1177/088307380201700303.
32) Nieves, AV (March 2002). Treatment of excessive daytime sleepiness in patients with Parkinson's disease with modafinil. Clin. Neuropharmacol. 25: 111–114. DOI:10.1097/00002826-200203000-00010.
33) a b Hart CL, Haney M, Vosburg SK, Comer SD, Gunderson E, Foltin RW (Jul 2006). Modafinil attenuates disruptions in cognitive performance during simulated night-shift work. Neuropsychopharmacology 31 (7): 1526–36. DOI:10.1038/sj.npp.1300991.
34) a b Henderson, David (April–June 2005). Modafinil-Associated Weight Loss in a Clozapine-Treated Schizoaffective Disorder Patient. Annals of Clinical Psychiatry; 17 (2): 95–97. DOI:10.1080/10401230590932407.
35) Efficacy and Safety of Modafinil Film-Coated Tablets in Children and Adolescents
36) Vaishnavi S, Gadde K, Alamy S, Zhang W, Connor K, Davidson JR (Aug 2006). Modafinil for atypical depression: effects of open-label and double-blind discontinuation treatment. J Clin Psychopharmacol 26 (4): 373–8. DOI:10.1097/01.jcp.0000227700.263.75.39.
37) Makris AP, Rush CR, Frederich RC, Kelly TH (Apr 2004). Wake-promoting agents with different mechanisms of action: comparison of effects of modafinil and amphetamine on food intake and cardiovascular activity. Appetite 42 (2): 185–95. DOI:10.1016/j.appet.2003.11.003.

COMITATO DI ETICA PER LA RICERCA BIOMEDICA
UNIVERSITA' DEGLI STUDI "G.D'ANNUNZIO"
AZIENDA SANITARIA LOCALE – CHIETI
E-mail: comitatodietica@unich.it

PROT. 2008/09 COET

L'anno duemilanove, il giorno venti, del mese di ottobre alle ore 14,30, presso l'Aula 5 del Nuovo Polo Didattico dell'Università "G. D'Annunzio" di Chieti, si è riunito il Comitato di Etica per la Ricerca Biomedica dell'Università /ASL di Chieti, formalmente istituito nel rispetto delle direttive europee della GCP DM 15.07.97 con deliberazione N° 7713/R del 21.07.94 e regolarmente accreditato dal Ministero della Salute.

Risultano presenti, assenti giustificati e assenti:

	Nominativo	Pres.	Giust.	Ass.
1	VACCA Michele	X		
2	NERI Matteo			X
3	NATOLI Clara		X	
4	CIPOLLONE Francesco			X
5	CONSOLI Agostino			X
6	D'ALONZO Luigi		X	
7	COTELLESE Roberto			X
8	DE BENEDETTO Fernando	X		
9	SALVATI Filippo	X		
10	ERRICHI Bruno Maria	X		
11	CARULLO Giuseppe	X		
12	MARINUCCI Riccardo	X		
13	CERULLI Aldo			X
14	BALLONE Enzo	X		
15	SCHIOPPA Francesco	X		
16	SCLOCCO Tonino	X		
17	STANISCIA Tommaso		X	
18	DI MASCIO Rocco	X		
20	PATRIGNANI Paola			X
21	CICCARELLI Renata	X		
22	BRUNETTI Lugi	X		
23	MELENA Ennio		X	
24	D'AMICO Rossana	X		
25	PACE Valeria	X		
26	DE ROSA Pier Luigi	X		
27	ALBANESE Paolo	X		
28	LEPORE Anna Raffaella		X	
29	SAVINO Carlo	X		
30	SEBASTIANELLI Raffaele	X		
31	ZAPPACOSTA Luigi			X
32	FARAONE Gabriele	X		
33	CASCAVILLA Michele			
34	RAZZOTTI Bernardo	X		
35	NARDONE Ginevra	X		
36	CIAMPOLI Rocco	X		
37	CIAVARELLI MACOZZI Sandro			X
38	MASCELLANTI Marco		X	

Il Presidente constatata la presenza del numero legale dichiara aperta la seduta.

<u>O M I S S I S</u>

3. Riesame protocolli:

OMISSIS

3.1 Protocollo "Raffronto, mediante tecnica fRMN delle mappe di attivazione/deattivazione cerebrale, dopo somministrazione di una dose singola di Modafinil 100mg". Studio spontaneo del Prof. Stefano Sensi c/o il Dipartimento di Scienze Mediche ed Applicate dell'Università "G. D'Annunzio " di Chieti;

Il Comitato etico esaminata la seguente documentazione pervenuta il 27/06/2009:

- Richiesta di autorizzazione a condurre lo studio al Dipartimento di scienze mediche di base e applicate;
- Protocollo di studio;
- Scheda raccolta dati;
- Scheda informativa e questionario per l'esecuzione dell'esame di risonanza;
- Dichiarazione di consenso informato;
- Dichiarazione di consenso informato per esame EEG;
- Nota informativa al medico curante;
- Dichiarazione di assunzione di responsabilità e garanzia;
- Prospetto dei costi;
- Elenco dei partecipanti alla sperimentazione;
- Curriculum Vitae dei partecipanti alla sperimentazione;

ed esaminato il protocollo emendato pervenuto in data 14/10/2009 in ottemperanza alle nostre richieste formulate nella seduta del 14/07/2009 esprime all'unanimità

parere favorevole alla conduzione dello studio.

Il Comitato richiede che venga informato dell'inizio dello studio, della sua conclusione, della sua eventuale interruzione con le motivazioni di essa.

Al Comitato Etico vanno inviati anche i rapporti sull'avanzamento dello studio, nonché il rapporto finale. Eventuali eventi avversi devono essere tempestivamente comunicati nei termini e con le modalità previste dall'art. 16 del Decreto Legislativo 24 giugno 2003, n. 211.

Si precisa inoltre che tutta la documentazione relativa allo Studio (comprese le schede raccolta dati e in particolare i moduli di consenso informato) dovrà essere archiviata e conservata a cura degli Sperimentatori per un tempo non inferiore agli anni 15. Tale documentazione dovrà essere prontamente reperita ed esibita in caso di controlli da parte delle autorità competenti (Ministero

della Salute, etc.). Nella corrispondenza si prega di citare sempre il protocollo e la data della seduta del Comitato Etico in cui è stata esaminata la documentazione.

<u>OMISSIS</u>

Null'altro essendovi da discutere o da deliberare la seduta è tolta alle ore 18,00.

Del che è verbale.

F.to IL PRESIDENTE

Prof. Michele Vacca

Università degli Studi "G. D'Annunzio" • COMITATO DI BIOETICA CHIETI

UNIVERSITA' DEGLI STUDI "G. D'ANNUNZIO"- ASL/CHIETI

COMITATO DI ETICA PER LA RICERCA BIOMEDICA

UFFICIO PRESIDENZA — **Allegato A) delibera n°** 2008/09 COET **del** 20/10/2009

PRESIDENTE	Prof. Michele Vacca	Preside Facoltà di Farmacia - Uni/ Chieti	

COMPONENTI

QUALIFICA-DISCIPLINA	TITOLARE	STRUTTURA	TITOLARE	STRUTTURA
Clinico	Matteo Neri, Clara Natoli, Francesco Cipollone, Agostino Consoli, Roberto Cotellese	Uni/Chieti		
Clinico	Fernardo De Benedetto, Filippo Salvati, Bruno Maria Errichi, Riccardo Marinucci, Raffaele Sebastianelli	Asl/Chieti		
Clinico	Luigi D'Alonzo, Marco Mascellanti	Membro esterno		
Biostatistico	Enzo Ballone, Tonino Sclocco, Francesco Schioppa	Uni/Chieti	Rocco Di Mascio<br>Tommaso Staniscia	Uni/Chieti
Farmacologo	Michele Vacca, Paola Patrignani	Uni/Chieti	Renata Ciccarelli<br>Luigi Brunetti	Uni/Chieti
Farmacista	Ennio Melena	Membro esterno	Rocco Ciampoli	Asl Chieti
Direttore Sanitario	Raffaella Lepore	Asl/Chieti	Carlo Savino	Asl/Chieti
Esperto in materia giuridica	Paolo Albanese	Asl/Chieti	Pier Luigi De Rosa	Membro esterno
Medicina Generale Territoriale in rappresentanza Ordine Medici	Valeria Pace, Gabriele Faraone	Ordine dei Medici		
Bioetica	Rossana D'Amico	Membro esterno		
Bioetica	Michele Cascavilla, Bernardo Razzotti	Uni/Chieti		
Infermieristico	Ginevra Nardone	Membro esterno	Luigi Zappacosta	Membro esterno
Associazionismo tutela malati	Giuseppe Carullo	Membro esterno	Aldo Cerulli	Membro esterno
Psicologo	Ciavarelli Macozzi Sandro	Membro esterno		

UFFICIO SEGRETERIA

Funzionario Amm.vo	Mauro Carabella	Asl/Chieti		
Amm.vo lib/prof.	Susanna Petaccia	Uni/Chieti		
